# Effectiveness of a questionnaire based intervention programme on the prevalence of arm, shoulder and neck symptoms, risk factors and sick leave in computer workers: A cluster randomised controlled trial in an occupational setting

**DOI:** 10.1186/1471-2474-11-99

**Published:** 2010-05-27

**Authors:** Erwin M Speklé, Marco JM Hoozemans, Birgitte M Blatter, Judith Heinrich, Allard J van der Beek, Dirk L Knol, Paulien M Bongers, Jaap H van Dieën

**Affiliations:** 1 Research institute MOVE, Faculty of Human Movement Sciences, VU University Amsterdam, Van der Boechorststraat 9, 1081 BT Amsterdam, the Netherlands; 2 Body@Work, Research Centre on Physical Activity, Work and Health, EMGO Institute, VU University Medical Center, Van der Boechorststraat 7, 1081 BT Amsterdam, the Netherlands; 3 Arbo Unie OHS, Daltonlaan 500, 3584 BK Utrecht, the Netherlands; 4 Department of Public and Occupational Health, EMGO Institute, VU University Medical Center, Van der Boechorststraat 7, 1081 BT Amsterdam, the Netherlands; 5 TNO Quality of Life, Polarisavenue 151, 2132 JJ Hoofddorp, the Netherlands; 6 Department of Clinical Epidemiology and Biostatistics, VU University Medical Center, De Boelelaan 1118, 1081 HZ Amsterdam, the Netherlands

## Abstract

**Background:**

Arm, shoulder and neck symptoms are very prevalent among computer workers. In an attempt to reduce these symptoms, a large occupational health service in the Netherlands developed a preventive programme on exposure to risk factors, prevalence of arm, shoulder and neck symptoms, and sick leave in computer workers. The purpose of this study was to assess the effectiveness of this intervention programme.

**Methods:**

The study was a randomised controlled trial. The participants were assigned to either the intervention group or the usual care group by means of cluster randomisation. At baseline and after 12 months of follow-up, the participants completed the RSI QuickScan questionnaire on exposure to the risk factors and on the prevalence of arm, shoulder and neck symptoms. A tailor-made intervention programme was proposed to participants with a high risk profile at baseline. Examples of implemented interventions are an individual workstation check, a visit to the occupational health physician and an education programme on the prevention of arm, shoulder and neck symptoms. The primary outcome measure was the prevalence of arm, shoulder and neck symptoms. Secondary outcome measures were the scores on risk factors for arm, shoulder and neck symptoms and the number of days of sick leave. Sick leave data was obtained from the companies. Multilevel analyses were used to test the effectiveness.

**Results:**

Of the 1,673 persons invited to participate in the study, 1,183 persons (71%) completed the baseline questionnaire and 741 persons participated at baseline as well as at 12-month follow-up. At 12-month follow-up, the intervention group showed a significant positive change (OR = 0.48) in receiving information on healthy computer use, as well as a significant positive change regarding risk indicators for work posture and movement, compared to the usual care group. There were no significant differences in changes in the prevalence of arm, shoulder and neck symptoms or sick leave between the intervention and usual care group.

**Conclusions:**

The effects of the RSI QuickScan intervention programme were small, possibly as a result of difficulties with the implementation process of the proposed interventions. However, some significant positive effects were found as to an increase in receiving education and a decrease in exposure to adverse postures and movements. With regard to symptoms and sick leave, only small and non-significant effects were found.

**Trial registration:**

Netherlands National Trial Register NTR1117

## Background

Arm, shoulder and neck symptoms, often referred to as RSI (Repetitive Strain Injury), are highly prevalent among computer workers [1]. A survey conducted amongst the working population in 15 European countries showed prevalences of 25% for neck/shoulder and arm pain, respectively [2]. A recent study amongst workers with these symptoms showed a symptom related decrease in the quality of life score of 31% [3]. To prevent symptoms, reduced performance and/or loss of production, employers implement interventions [4]. In the Netherlands, 61% of the organisations with more than 100 employees implemented such interventions [5]. Nevertheless, few randomised controlled trials, with sufficient size and statistical power, have been conducted [6] and, consequently, knowledge about the effectiveness of frequently used interventions is still lacking.

Various ergonomic, psychosocial and organisational risk factors for arm, shoulder and neck symptoms have been suggested [7] and the onset of such symptoms might be caused by a combination of these factors. However, intervention studies are often aimed at one specific work-related factor, such as the duration of computer work, or mouse use, the use of an adjustable chair, or at single work-related psychosocial factors, such as insufficient recovery time and insufficient social support [8-11]. Even though moderate evidence for some interventions was found, no strong evidence for the effectiveness of interventions in reducing symptoms in occupational settings could be established [8-11]. The fact that these interventions are usually aimed at the general population of computer workers and not at specific workers with a high exposure to certain factors, or that selected interventions did not address the most prominent risk factors might contribute to a limited effectiveness. Prevention efforts should ideally be targeted at specific populations with a high risk [12], as reducing a low exposure even further is unlikely to yield much effect. To improve the effectiveness of interventions aimed at reducing the prevalence of arm, shoulder and neck symptoms, exposure to risk factors, and sick leave in computer workers, a multidimensional intervention programme was developed. This intervention program is quite unique in that it incorporates many different aspects, addressing a broad spectrum of potential risk factors. Instead of using generic strategies, which is common among occupational health services in the Netherlands, this method establishes a risk profile of the target population and subsequently advises interventions following a decision tree based on that risk profile.

The objective of this study was to assess the effectiveness of this intervention programme on the prevalence of arm, shoulder and neck symptoms, reduction of exposure to risk factors, and sick leave in a population of computer workers.

## Methods

### Design and study population

**Figure 1:**
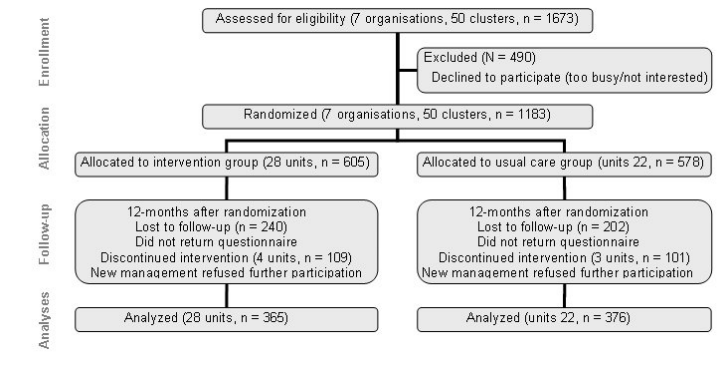
**Flow of clusters and participants through the phases of the trial**. Units that discontinued the intervention, did receive full feedback on their RSI QuickScan questionnaire results, but declined other proposed interventions as a result of financial constraints.

The study was designed as a cluster Randomised Controlled Trial (RCT) with an intervention group and a usual care group. Participants were recruited from January 2005 to January 2006. The participating organizations were approached through the occupational health service and selected by willingness to participate. All workers of participating organisational units were invited to participate by e-mail. Measurements took place at baseline, after 6-months and after 12-months. The source population consisted of computer workers from 7 Dutch organisations in various branches (e.g. health care, local government, nature conservation, engineering, education and regulatory affairs), located throughout the Netherlands. The population consisted of office staff, local government officials, engineers, consultants, teachers, health care personnel, nature conservation professionals, researchers and managers. Figure 1 shows the CONSORT [13] diagram of the flow of clusters and participants through the phases of the trial. Of the 1,673 persons who were invited to participate in the study, 1,183 persons (71%) completed the baseline questionnaire. A total of 741 persons participated at baseline as well as at 12-months follow-up and were included in the analyses. Units that discontinued the intervention, did receive full feedback on their questionnaire results, but declined other proposed interventions as a result of financial constraints. Prior to inclusion, all participating organisations expressed their willingness to take preventive measures in case the results of the investigation would give cause to this. Employees were given time during work to fill out the questionnaires and participate in the interventions. Workers with and without arm, shoulder and neck symptoms were included. Workers had to be able to read Dutch, such that they could understand the information provided and complete the questionnaire. The study design, protocols, procedures and informed consent form were approved by the Ethics Committee of the Faculty of Human Movements Sciences of the VU University Amsterdam, and all participants electronically provided informed consent before filling out the baseline questionnaire.

### Randomisation

The participants were assigned to either intervention group or usual care group by means of cluster randomisation. To prevent unbalanced randomisation, workers were pre-stratified by the HRM departments of the participating organisations, who formed clusters of approximately the same size and with a comparable amount of computer work. Teams or departments were left intact, to avoid crossover effects and to enhance the compliance in the intervention group. In some cases, clusters consisted of participants in the same building or floors of a building. Allocation concealment was performed by using sealed envelopes containing the names of the clusters in each organisation. The envelopes were then randomly divided into an intervention and usual care groups by the HRM department. Even though participants were not informed about their allocation, workers could not be blinded for the intervention due to the character of the interventions. The principal investigator was not blinded for group allocation when performing the data analysis.

### Data collection

At baseline and 6 and 12 months follow-up, the workers completed the internet-based RSI QuickScan questionnaire on exposure to risk factors and the 6-months and 7-days prevalence of arm, shoulder and neck symptoms. The psychometric properties of this measurement tool have previously been tested and results indicate an acceptable reliability, concurrent validity and homogeneity[7]. All participants received an e-mail in which: 1) the goal of the investigation was explained, 2) information was provided on protection of confidentiality and 3) individual login information was presented. A letter with information about the study was attached to this e-mail. An incentive was allotted amongst workers who participated in all measurements for each organisation. A description of the content of the questionnaire can be found at Additional file 1: Questionnaire http://www.rsiquickscan.com/research/questionnaire.pdf.

Participants who had not logged in to the RSI QuickScan and those who did log in but did not complete the questionnaire received a reminder to complete the questionnaire two weeks after the first e-mail and again one week thereafter. One week after the final reminder (one month after the initial mail was send) access to the online questionnaire was closed.

### Intervention group

**Figure 2:**
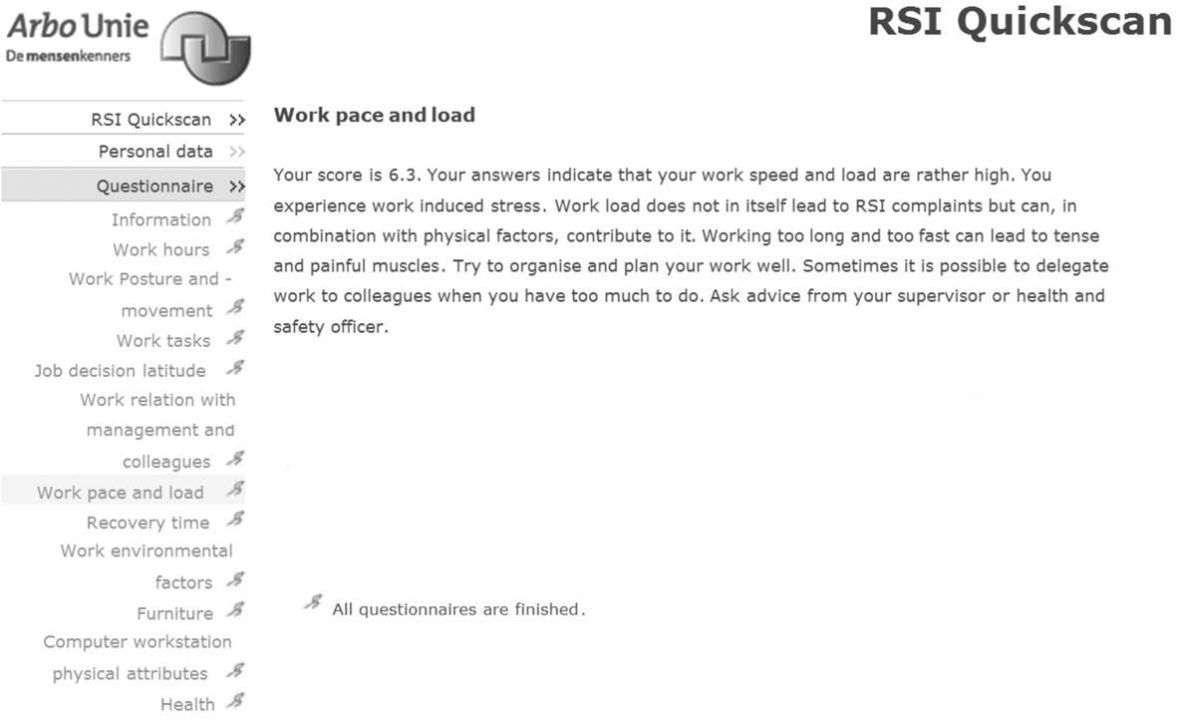
**An example of the individual feedback on the questionnaire results**.

**Figure 3:**
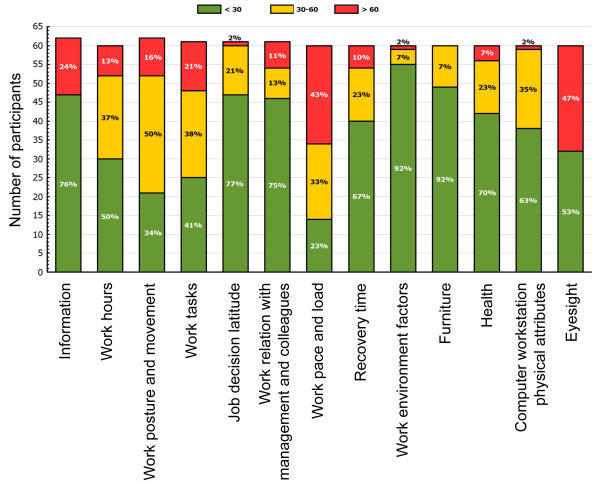
**An example of the feedback on the questionnaire results of a group**.

The intervention group received full feedback on their RSI QuickScan questionnaire results. This feedback was given after completing each section of the questionnaire and consisted of scores on a scale from 1 to 10, a visual representation of the score with a graph, an interpretation of the score and an elaborate advice on the specific actions that they could personally take in order to reduce their risk of arm, shoulder and neck symptoms (Figure 2). If workers reported severe symptoms in the arm, shoulder and neck region, their occupational physician invited them for a consultation. Furthermore, from the information given by the respondents, a risk profile was made, using the traffic light coding system, also known as the RAG rating [14]. A score of 30% or less of the maximum on a scale was classified as a low risk, colour coded "green". A score of 31% to 60% of the maximum on a scale was classified as a medium risk, colour coded "amber". A score of 61% or more of the maximum on a scale was classified as a high risk, colour coded "red". All scales combined in a graph illustrate the risk profile of an individual or a group. This graph was provided not only for the individual, but also for the organisation, the department or function group (Figure 3). If more than 30% of the participants had a red score or more than 60% of the participants had a red or amber score, a tailor-made intervention programme was proposed. Per scale a (set of) intervention(s) to be advised to the participating organisations was pre-defined.

The interventions were aimed at each of the factors in the RSI QuickScan, with a total of 16 interventions aimed at reducing the associated risk. Examples of proposed interventions are:

• Individual level

◦ Individual Workstation Check - An advisor visits the worker at his/her work station and advises on ergonomic aspects, such as the set-up of the workstation and the furniture.

◦ Eyesight check - in order to determine whether there is a need for computer glasses.

◦ A visit to the occupational health physician

• Group level

◦ Education programme on the Prevention of arm, shoulder and neck symptoms for Employees - This involves education about arm, shoulder and neck symptoms, the ergonomic aspects of the workstation and the effects of work organisational factors.Developing

◦ Handling Stress in the Workplace - A training aimed at getting insight into stress and stress situations, to improve coping, learn relaxation techniques and influence one's own work situation.

To give the organisations a choice in intensity and costs of interventions, multiple interventions were available, differing mainly in duration, ranging from a two-hour information session to a training consisting of eight half-day sessions. Interventions were carried out both on an individual and a group level. Depending on their risk profile, some workers were offered multiple interventions. The organisations are responsible for carrying out the program. However, the quality control of interventions lies with the Occupational Health Service, whose quality is certified by the Ministry of Social Affairs and Employment, and the professionals who work for it. A description of all interventions can be found at Additional file 2: Interventions http://www.rsiquickscan.com/research/interventions.pdf. There were no harmful effects of the interventions observed for the individuals during the study.

### Usual care group

In contrast to the intervention group, the usual care group did not receive elaborate advice on the actions that they could personally take after completing the RSI QuickScan, but more general and limited advice. Furthermore, they did not receive interventions based on the risk profile during the time of the study. However, because of ethical considerations, workers, who reported severe symptoms in the arm, shoulder and neck region were also invited by their occupational physician for a consultation, even though they were part of the usual care group. These workers were treated according to the Dutch guideline on arm, shoulder and neck symptoms [15], which states that workers should try to continue their work, except for tasks that induce severe pain. Furthermore, they received advice on possible treatments, adjustments in the workplace and could be referred to a physical therapist. For other actions the usual care group was put on a waiting list, so that they received interventions that were similar to those in the intervention group, but only after the study was ended.

### Outcome measures

The primary outcome measure was the prevalence of arm, shoulder and neck symptoms. Secondary outcome measures were the scores on risk factors for arm, shoulder and neck symptoms and the number of days of sick leave.

To assess exposure to potential risk factors for the establishment of risk profiles related to arm, shoulder and neck symptoms in computer workers we used a questionnaire, consisting of items on work (e.g. work hours, work tasks), relation with management and colleagues, office ergonomics (e.g. furniture and computer workstation physical attributes) and health (e.g. arm, shoulder and neck symptoms). In total, the questionnaire consisted of 95 items. Reliability and concurrent validity were shown to be satisfactory [7].

The prevalence of arm, shoulder and neck symptoms was estimated with the questionnaire, which used a slightly altered version of the Nordic Questionnaire by Kuorinka et al. [16]. It specifies 7 areas in the arm, shoulder and neck region, as suggested by Sluiter et al. [17]. Furthermore, the questionnaire did not only show a dorsal view of the arm, shoulder and neck region, but also a frontal view. The Nordic Questionnaire has been extensively tested for validity [16].

The prevalence of arm, shoulder and neck symptoms was defined as: regular or long-lasting symptoms in one or more regions of arm, shoulder and neck, in the past six months and/or in the past seven-days. A description of these questions can be found at Additional file 1: Questionnaire http://www.rsiquickscan.com/research/questionnaire.pdf. The overall prevalence of arm, shoulder and neck symptoms was divided into two subgroups: proximal (neck, upper-back and shoulders) and distal (elbows, forearms, wrists and hands) symptoms. The total symptom score consisted of the sum of points scored on the scales arm, shoulder and neck symptoms. Information on the number of days of sick leave was obtained from the HRM departments of the participating organisations. The data consisted of total sick leave, maternity leave excluded, and not solely sick leave due to (serious) arm, shoulder and neck symptoms.

### Statistical analysis

In the sample size calculation, an intracluster correlation of 0.05 was assumed, an average of 15 workers per cluster, an initial participation of 70%, and a loss to follow-up of 40%. Under these assumptions, we anticipated to be able to detect a difference of 15% (35% versus 50%) in the prevalence of symptoms between the intervention and usual care group (power of 80%; one-sided significance level, 0.05) with 225 workers with completed questionnaires in 25 clusters assigned to both the intervention and control group [18].

Only workers who filled out the baseline questionnaire and the 12-months follow-up questionnaire were included in the analyses. Data of the 6-months follow-up were not analyzed, as only few interventions were implemented prior to this measurement. Analyses to estimate the effect of the intervention were pre-specified and done according to the intention-to-treat principle [19].

Multilevel analyses were used to investigate the differences in changes in outcome variables regarding prevalence of arm, shoulder and neck symptoms, risk factors, and sick leave between the intervention group and the usual care group after 12 months of follow-up. In the regression model the values of the outcome variables, either continuous or dichotomous, at 12-months follow-up were considered as dependent variables. The intervention level (yes/no) and the baseline values of the outcome variables were considered as independent variables, so that scores at 12 months follow-up were corrected for baseline. For the dichotomous outcome variables, i.e., information, eyesight, prevalence of (overall, proximal and distal) arm, shoulder and neck symptoms, logistic multilevel regression analysis were used. No other corrections were performed.

Multilevel analyses were used in order to adjust for possible dependence between observations from the same organisation or department. The data of this study were clustered at three levels: company, department, and individual. All multilevel statistical analyses were performed using MLwiN version 2.02 [20]. All non-multilevel statistical analyses were performed using SPSS version 16 [21].

## Results

### Randomisation

There were no significant differences regarding age, gender, working more than 30 hours per week, and working more than 4 hours with the computer per day, between the intervention and usual care groups (Table 1). However, there were significant differences between the two groups in the number of sick leave days in the six months prior to baseline, with the usual care group reporting more sick leave days.

**Table 1:** Baseline characteristics of the study population for both groups.

	Usual care (*N *= 578)	Intervention (*N *= 605)
Gender (male)	334 (57.8%)	378 (62.5%)
Age (years) (mean, SD)	43.8 (9.7)	44.4 (9.2)
Work more than 30 hours per week	445 (76.9%)	452 (74.6%)
Work more than 4 hours per day with the computer	406 (70.3%)	430 (70.0%)
Number of sick leave days in the 6-months period prior to baseline (median)	1.0	0.0
- % workers with 0 days sick leave	49	50
- % workers with 1 - 7 days sick leave	27	30
- % workers with 7 - 21 days sick leave	15	14
- % workers with > 21 days sick leave	9	6

Values are numbers (percentages) unless stated otherwise. SD = standard deviation

### Non-response analysis

Respondents and non-respondents were similar in age, but there were significantly fewer men in the respondent category. Non-respondents at follow-up had significantly higher risk scores on the scales "information", "work posture and movement", and "furniture". Non-respondents had a lower prevalence of distal symptoms, but a significantly higher number of sick leave days in the 6-months period prior to baseline.

### Utilization rate interventions

Of the 16 possible interventions 6 were implemented (utilization rate in the intervention/usual care group % yes): Occupational health physician (8/6), Education on the Prevention of RSI for Employees (26/0), RSI and Stress (24/2), Eyesight check (19/7), Individual Workstation Check (2/1), Task analyses (1/0).

### Effect of the intervention programme

Table 2 gives an overview of the risk factors, prevalence of arm, shoulder and neck symptoms and sick leave at baseline and 12 months of follow-up. After 12-months of follow-up, the intervention group scored significantly better than the usual care group on the scales "Information" and "Work posture and movement" (Table 3). Corrected for baseline values, a significant Odds Ratio of 0.48 (95% CI: 0.28 to 0.82) was found for information, indicating that at follow-up the participants in the intervention group had a two times higher chance to have had information concerning prevention than the usual care group. For the scale work posture and movement, the significant regression coefficient of -0.35 (95% CI: 0.68 to -0.03) indicates that the intervention group had at follow-up on average 0.35 points less on a 0 to 11 points scale than the usual care group, indicating a slightly lower risk. The results were corrected for baseline values. There was a slight reduction in scores for several other factors, but this occurred in both, the intervention and usual care, groups. There were no significant differences in the changes in prevalence of arm, shoulder and neck symptoms between the intervention and usual care group. The overall prevalence of arm, shoulder and neck symptoms decreased by 9% in both the intervention (decrease from 51% to 42%) and usual care group (decrease from 56% to 47%). There were no significant differences in changes in sick leave between the intervention and usual care group. Compliance of the participants varied from 51%, for an eyesight check, to 89% for a visit to the occupational health physician. Low compliance was sometimes caused by the decision of participating organisations not to accept (parts of) the proposed intervention plan. In two of the participating organisations new management decided not to implement any of the proposed interventions.

**Table 2:** Risk factors, symptoms and sick leave at baseline and follow-up.

	Usual care		Intervention	
Risk factors	Baseline	12-month	Baseline	12-month
Information (range 0-1)				
(% workers with a red score)	40	25	41	17
Work hours				
(median; range 0-12)	4.0 (52.3%)	4.0 (51.3%)	4.0 (51.5%)	4.0 (50.8%)
(% workers with a red/amber score)	11/30	8/31	11/29	8/30
Work posture and movement				
(median; range 0-11)	4.0 (62.5%)	3.0	4.0 (64.4%)	2.0
(% workers with a red/amber score)	13/40	9/26	11/41	5/28
Work tasks				
(median; range 0-5)	1.0 (53.3%)	1.0 (58.2%)	1.0 (53.8%)	1.0 (60.7%)
(% workers with a red/amber score)	15/31	11/30	14/32	12/27
Job decision latitude				
(median; range 0-9)	0.0 (52.7%)	0.0 (56.8%)	0.0 (55.1%)	0.0 (57.9%)
(% workers with a red/amber score)	2/15	2/13	3/11	3/10
Work relation with management and colleagues (median: range 0-7)	1.0 (65.5%)	0.0 (62.2%)	1.0 (59.0%)	0.0 (60.9%)
(% workers with a red/amber score)	7/17	9/15	8/18	13/15
Work pace and load				
(median; range 0-8)	4.0	3.0 (52.4%)	3.0	3.0 (54.3%)
(% workers with a red/amber score)	37/26	34/28	34/31	34/28
Recovery time				
(median; range 0-6)	1.0 (74.3%)	0.0	1.0 (75.0%)	1.0
(% workers with a red/amber score)	6/19	6/18	6/19	7/18
Work environmental factors				
(median; range 0-4)	1.0 (76.4%)	1.0 (77.8%)	1.0 (76.4%)	1.0 (81.2%)
(% workers with a red/amber score)	7/17	7/15	5/19	4/14
Furniture				
(median; range 0-10)	2.0 (63.1%)	1.0	2.0 (66.4%)	2.0
(% workers with a red/amber score)	5/32	1/25	4/30	3/28
Computer workstation physical attributes (median; range 0-7)	1.0 (49.3%)	1.0 (63.5%)	1.0 (51.3%)	1.0 (66.9%)
(% workers with a red/amber score)	2/22	0/14	3/21	1/11
Eyesight (range 0-1)				
(% workers with a red score)	34	29	37	29
**Neck, shoulder and arm symptoms**				
Prevalence symptoms (%)	56	47	51	42
Prevalence proximal symptoms (%)	46	38	39	31
Prevalence distal symptoms (%)	28	24	31	24
Total symptom score				
(median; range 0-44)	7.0	6.0	7.0	6.0
(% workers with a red/amber score)	12/24	9/19	9/21	4/20
**Sick leave**				
Number of sick leave days (median)	1.0 (52.2%)	1.0 (51.7%)	0.0 (50.2%)	0.0 (50.9%)
- % workers with 0 days sick leave	49	45	50	51
- % workers with 1 - 7 days sick leave	27	31	30	29
- % workers with 7 - 21 days sick leave	15	15	14	12
- % workers with > 21 days sick leave	9	9	6	8

Values indicate the mean except where otherwise indicated. Where applicable, scale extremes are given in the left-hand column. High scores reflect a high risk. (%) = cumulative percent at median value.

**Table 3:** Results of the longitudinal multilevel analyses.

	Intervention effect β/Odds (95% C.I.)	
Risk factors	β	Odds
Information (0/1)	.	0.48 (0.28; 0.82)
Work hours (range 0-12)	-0.08 (-0.33; 0.17)	.
Work posture and movement (range 0-11)	-0.35 (-0.68; -0.03)	.
Work tasks (range 0-5)	-0.04 (-0.24; 0.17)	.
Job decision latitude (range 0-9)	-0.10 (-0.35; 0.15)	.
Work relation with management and colleagues (range 0-7)	0.02 (-0.34; 0.38)	.
Work pace and load (range 0-8)	-0.00 (-0.31; 0.30)	.
Recovery time (range 0-6)	0.05 (-0.16; 0.25)	.
Work environmental factors (range 0-4)	-0.09 (-0.24; 0.07)	.
Furniture (range 0-10)	0.24 (-0.12; 0.61)	.
Computer workstation physical attributes (range 0-7)	-0.09 (-0.29; 0.10)	.
Eyesight (0/1)	.	0.88 (0.62; 1.27)
**Arm. shoulder and neck symptoms**		
Prevalence arm, shoulder and neck symptoms	.	0.89 (0.61; 1.30)
Prevalence proximal symptoms	.	0.78 (0.54; 1.12)
Prevalence distal symptoms	.	0.90 (0.49; 1.67)
Total symptom score (range 0-44)	-0.75 (-1.78; 0.29)	.
**Sick leave**		
Days of sick leave	-0.27 (-2.85; 2.31)	.

β, regression coefficient; 95% C.I., 95% confidence interval.

The results show the differences in changes in outcome variables regarding risk factors, the prevalence of arm, shoulder and neck symptoms and the amount of sick leave between the intervention group and the usual care group after 12 months of follow-up. The results were corrected for baseline. β represents the difference in score for the range indicated in the table.

## Discussion

The overall effects of the RSI QuickScan intervention programme were small. The overall prevalence of arm, shoulder and neck symptoms decreased by 9% in both the intervention and usual care group, but no significant differences between groups was found. The positive findings of this study were a significant improvement of the intervention group on the scales "Information" and "Work posture and movement" after 12 months. There were no significant differences in sick leave between the intervention and usual care groups.

### Comparison with other studies

These findings regarding the effectiveness of this intervention programme on the reduction of prevalence of arm, shoulder, neck symptoms, exposure to risk factors, and sick leave in computer workers, partially confirm those of previous studies, where also small effects were found. Several studies on the effectiveness of preventative interventions have been published in the last decade. A systematic review by Brewer et al [9] found moderate evidence for no effect of workstation adjustments and also no effect of rest breaks together with exercise during the breaks. However, the review did find a positive effect of alternative pointing devices on musculoskeletal outcomes. A review by Boocock et al [8] identified no single-dimensional or multi-dimensional strategy for intervention that was considered effective across occupational settings. It is important to note that no study comparable to the RSI QuickScan intervention programme, where the advised set of interventions is based on a previously established risk profile, was found in the literature.

### Implementation of the interventions

The limited effect of the RSI QuickScan intervention programme might be caused by problems with regard to the implementation of the interventions. The interventions were sold at their normal commercial price and even though all participating organisations prior to inclusion had stated that they were prepared to invest in the necessary preventive measures, in practice some of the participating organisations chose not to do so, due to a low degree of support from the management and/or lack of financial resources. Consequently, workers who should have participated in an intervention were never offered one, let alone participated in one. The intention was to start the interventions that were accepted within a three month period after the first measurement was finished. In practice, some of the interventions started after 6-months, leaving little time for effects on the arm, shoulder and neck symptoms or sick leave.

### Effectiveness of the interventions

Exposure to most risk factors and prevalence of arm, shoulder and neck symptoms decreased in both groups. The information on risk factors provided in the questionnaire and the feedback seems to have led to more favourable behaviour and therefore, a decrease in risk factors. Furthermore, the focus on arm, shoulder and neck symptoms in the participating companies may also have caused greater awareness of the risks attributed to computer use and may have contributed to the overall decline of risk factors and arm, shoulder and neck symptoms. These positive effects in both the intervention and the control group may have made it more difficult to achieve results op top of this, which would make it harder to detect significant differences between the intervention and usual care group. The questionnaire contains questions about the duration and location of the symptoms, but it does not ask questions about the pain intensity and function, which might make it more difficult to find an effect.

The usual care group had an overall higher prevalence of arm, shoulder and neck symptoms compared to the intervention group and the symptoms in the usual care group were more serious. This makes a regression to the mean likely to occur.

The data we obtained from the HRM department consisted of total sick leave and not solely sick leave due to (serious) arm, shoulder and neck symptoms. Since the average number of sick leave days at baseline was already relatively low and considering that sick leave due to arm, shoulder and neck symptoms was even smaller, the probability to find a significant decrease was low.

Even though arm, shoulder and neck symptoms are highly prevalent among computer workers, it remains to be seen if long-lasting pain is related to elements of computer use[22,23]. This might be another reason for the negative findings in this study.

### Strengths and limitations of the study

Strengths of this study are its solid RCT design, combined with cluster randomisation to minimize contamination between the two groups and to increase compliance with the interventions. Comparability between the groups was good. The intervention took place during a full year eliminating possible seasonal variance, which could have biased the results. Furthermore, the size of the research population in the present study provided sufficient statistical power. Generalisation of the results to other computer workers is enhanced by the fact that the research population consisted of computer workers from all over the Netherlands, employed in different settings (e.g. health care, local government, engineering, education) and a broad range of jobs. The age and gender distributions corresponded with the distribution in the working population in the Netherlands.

There are also some limitations within this study. Since the respondents, in comparison to the non-respondents at baseline, had received more information, a better work posture and better furniture, there was less room for improvement in these areas, which might have had a negative effect on the results. Also the fact that they had a significantly lower number of sick leave days in the 6-months period previous to baseline makes it more difficult to get positive results.

The test-retest validity, concurrent validity and homogeneity of the RSI QuickScan have been studied and the RSI QuickScan has proven to be a valid instrument to assess risk factor and arm, shoulder and neck symptoms. However, the reliability of self-reported duration of computer use and postural load, by means of questionnaires, has been questioned [24-28]. Furthermore, there is still uncertainty about risk factors and hence, the predictive validity of the RSI QuickScan is unsure.

## Conclusions

In conclusion, the positive effects of the RSI QuickScan intervention programme are limited to a reduction of exposure to only some risk factors. No significant effects were found for most risk factors, for arm, shoulder and neck symptoms and sick leave. This might be caused by the fact that the population consisted of computer workers with- and without symptoms, and by workers not receiving the advised intervention. For those who did receive an intervention, the duration and intensity of the interventions was often low. Given the still high percentage of workers suffering from arm, shoulder and neck symptoms, further studies on the effect of interventions in reducing arm, shoulder and neck symptoms in occupational settings are recommended.

## Competing interests

The first co-author is an employee of Arbo Unie. This non-profit occupational health service is the proprietor of the RSI QuickScan. The study was granted by the Netherlands Organization for Health Research and Development (ZonMw) and the Foundation Arbo Unie Netherlands.

## Authors' contributions

ES participated in the design, performed the study, including statistical analysis and drafted the manuscript. MH and JvD participated in the design, helped with the statistical analysis and drafting the manuscript. BB, JH, AvdB and PB participated in the design of the study, reviewed the paper and made corrections. DLK helped with the statistical analysis, reviewed the paper and made corrections. All authors read and approved the final manuscript.

## Pre-publication history

The pre-publication history for this paper can be accessed here:

http://www.biomedcentral.com/1471-2474/11/99/prepub

## Supplementary Material

Additional file 1 Questionnaire. Click here for file

Additional file 2 Interventions. Click here for file
