# Supplementary material for: Effectiveness of a questionnaire based intervention programme on the prevalence of arm, shoulder and neck symptoms, risk factors and sick leave in computer workers: A cluster randomised controlled trial in an occupational setting
Source: BMC Musculoskelet Disord. 2010 May 27;11:99. doi: 10.1186/1471-2474-11-99 (PMC2890602; doi:10.1186/1471-2474-11-99)
Supplement: Additional file 1 — Questionnaire. [file 1471-2474-11-99-S1.PDF]

Main areas	Scales and questions
Work	- Information

1. information on computer work and health

- Work hours

2. - amount of work hours per week
3. - amount of work days per week
4. - hours computer work per day
5. - hours private computer use per day
6. - amount of breaks per day
7. - total break time per day

- Work posture and movement

8. - upper body slightly bent forward
9. - upper body bent forward a lot
10. - trunk slightly twisted
11. - trunk twisted a lot
12. - upper body bent forward and twisted
13. - neck hunched forward
14. - neck hunched backward
15. - neck twisted
16. - wrist bent
17. - wrist extended
18. - wrist twisted

- Work tasks

19. - repetitive movements arm. hand fingers
20. - repetitive twisting/bending upper body
21. - repetitive twisting/bending upper body
22. - same work all day
23. - same work every day
24. - repetitive movements

- Job decision latitude

25. - choose time begin/stop work
26. - choose time breaks
27. - choose which days off
28. - choose how to do your work
29. - choose order of work tasks
30. - choose when work tasks
31. - leave workspace
32. - choose stop work
33. - control work pace

- Work relation with management and colleagues

34. - good management
35. - irritated by others
36. - management notes what you say
37. - good general atmosphere
38. - management knows you / your work
39. - support direct supervisor
40. - support colleague
41. - sufficient information from company

- Work pace and load

42. - pace of work / work load regularly high
43. - regularly work under time pressure
44. - hurry to finish on time
45. - regularly problems pace work / work load
46. - should take it easier
47. - work too tiring
48. - have to work very fast
49. - a tremendous amount of work
50. - enough time to finish work

- Recovery time

51. - feel mentally exhausted
52. - feel empty after a days work
53. - feel tired when waking up in the morning
54. - feel 'burned out'
55. - feel frustrated by job
56. - feel work asks too much
57. - feel at the end of your tether

Office ergonomics

- Work environment factors

58. - bothered by light from outside
59. - bothered by reflection in your monitor
60. - cold. draughts or changes in temperature
61. - disturbed by noise

- Furniture

62. - correct height chair
63. - comfortable chair
64. - correct height arm rests
65. - adjustable width arm rests
66. - correct length arm rests
67. - correct height desk
68. - adjustable height desk
69. - sufficient work surface
70. - sufficient leg room
71. - availability footrest

- Computer workstation physical attributes

72. - availability external mouse and keyboard
73. - hindered by length mouse cable
74. - mouse works properly
75. - document holder available
76. - head set
77. - correct height monitor
78. - correct viewing distance monitor

- Eyesight

79. - eye complaints (problems focusing, burning- or watery eyes)?

Health - **Prevalence of arm, shoulder and neck symptoms**

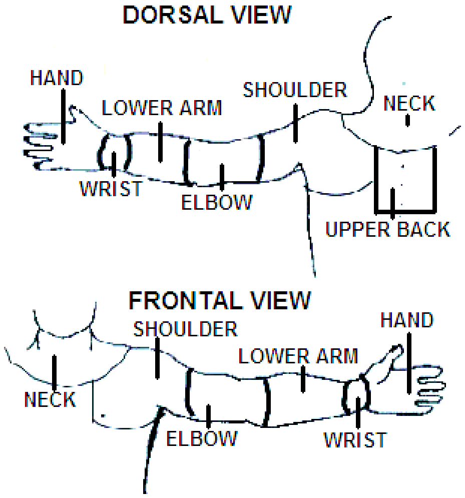

- pain or discomfort in the last **6 months** in:
  80. - neck
  81. - upper back
  82. - shoulder
  83. - elbow
  84. - lower arm
  85. - wrist
  86. - hand

- pain or discomfort in the last **7 days** in:
  87. - neck
  88. - upper back
  89. - shoulder
  90. - elbow
  91. - lower arm
  92. - wrist
  93. - hand

-
  94. - pain or discomfort linked to work?
  95. - pain or discomfort developed during work?
