# Supplementary material for: Effectiveness of a questionnaire based intervention programme on the prevalence of arm, shoulder and neck symptoms, risk factors and sick leave in computer workers: A cluster randomised controlled trial in an occupational setting
Source: BMC Musculoskelet Disord. 2010 May 27;11:99. doi: 10.1186/1471-2474-11-99 (PMC2890602; doi:10.1186/1471-2474-11-99)
Supplement: Additional file 2 — Interventions. [file 1471-2474-11-99-S2.PDF]

## INTERVENTIONS

### Individual Workstation Check

With a one-time visit, the workstation and work behavior will be assessed with the aid of the 5W's (work tasks, work periods, work pressure, workstation and working method) model. The advisor will pay special attention to ergonomical aspects such as the set-up of the workstation, the furniture, work posture and work organizational factors etc. The advisor will give technical en behavioral advice and will include these in a written report.

### Training On-the-Job

As a supplement to the one-time guidance of the workstation check and with the accent on work posture and work techniques of the employee, "training on the job" will be done in order to achieve a better and permanent effect. During this training, feedback tools are used such as photo or video equipment and/or a muscle tone measurement device.

### RSI Counselling Sessions

Low threshold, walk-in counselling will take place within your organization, aimed at prevention, for those employees that have complaints or questions about RSI. The counselling will be done by an Occupational Health Physician or an Occupational Physical Therapist. Depending on the situation, the counselling may consist of a physical check and/or a workstation orientation (with tips and exercises).

### Training Course Assertiveness and Negotiation

This training is for supervisors and employees to develop an assertive attitude in their negotiation skills, so that negotiations can be conducted in an effective and pleasant manner. The training takes three half-days.

### Individual Coaching

Individual coaching is suitable for employees of all levels. Insight is given into one's own skills and capacities and one learns how to better apply them.

### Time Management Course

A course designed for employees who want to learn how to deal with stress situations resulting from a high work tempo and heavy work load. As organizations become less hierarchic, it becomes more and more important to be able to manage your own time because no one tells you what to do and when. During the course "Time Management" one learns especially how to set priorities and stick to them.

### Stress Management for Supervisors

Training for supervisors which takes at least three half-days that will enable them to recognize stress signals from employees at an early stage and to communicate about this in a responsible manner on individual and departmental levels.

### Handling Stress in the Workplace Course

A training of eight half-days which is aimed at getting insight into stress and stress situations, to improve one's own resistance, learn relaxation techniques and have influence on one's own work situation.

**Initiating RSI Policy**

Advice will be given for a good RSI policy. Often this involves an approach to RSI that takes several years. The Arbo Unie advisor supports you during the development and the execution stage of that policy.

**Education for the Prevention of RSI for Employees**

This involves education for groups of employees about RSI, the ergonomical aspects of the workstation and the cause and results of over-burdening. The informative sessions are set up to be practicable, i.e. learning correct work behavior in one's own work situation. Feedback is done through photo or video recordings; the responsibility of employees themselves will be stressed. In addition, ergonomical improvement possibilities will be discussed.

**RSI Education for Supervisors**

Your supervisors will learn the risk factors for RSI and how employees with RSI can be guided. Not only the physical ergonomical aspects of RSI but also the psychological risk factors are covered and how to deal with this on an individual level.

**RSI and Stress**

A special training for supervisors and employees about RSI as an organizational problem, the causes of RSI and stress and "coping" mechanisms will be covered extensively. The training will be given by an ergonomist and a Personnel and Organization expert.

**Computer Work Expert Course (train the trainer)**

Aimed at ensuring ergonomical workstations, correct work behavior and correct work posture within a company or organization. After the course, the internal trainer/coach will be able to optimally adjust a workstation for an employee, demonstrate the correct work techniques for present and future employees and give training. He/she will be able to recognize sticky points, correct situations, offer policy options and deal with resistance.

**Training Team Building**

A training of several half-days with the object to improve the team by making action plans together, learning how to deal with differences and obtaining increased effectiveness.

**Team Coaching**

Team coaching can contribute to the following: learning to optimally use each others qualities, improving cooperation, increasing productivity of the team and being more flexible and decisive in the face of changes. Team Coaching may be used as a separate tool as well as a continuation of the Team Building in order to coach the implementation of the action plans.

**Communication and Co-operation**

A practical training of two half-days for supervisors and employees which deals with the basic elements of effective communication.
